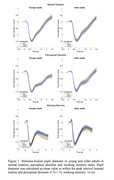# Pupil dilation as specific marker of cognitive effort in the memory domain: an eye‐tracking study in younger and older adults

**DOI:** 10.1002/alz70861_108810

**Published:** 2025-12-23

**Authors:** Carina Zeitler, Sabrina Lenzoni, Joshua Horngacher, Jamie Kofler, Philipp Feistimantl, Ophir Gat, Dorothea Hämmerer

**Affiliations:** ^1^ University of Innsbruck, Innsbruck, Tyrol Austria; ^2^ Institute of Cognitive Neuroscience, University College London, London UK; ^3^ Institute of Cognitive Neurology and Dementia Research (IKND), Otto‐von‐Guericke University, Magdeburg Germany; ^4^ Wellcome Centre for Human Neuroimaging, Queen Square Institute of Neurology, University College London, London UK; ^5^ Center for Behavioral Brain Sciences, Magdeburg Germany; ^6^ German Center for Neurodegenerative Diseases, Magdeburg Germany; ^7^ Wellcome Centre for Human Neuroimaging, University College London (UCL), Queen Square Institute of Neurology, London UK; ^8^ Institute of Cognitive Neurology and Dementia Research, Otto‐von‐Guericke University Magdeburg, Magdeburg Germany

## Abstract

**Background:**

Pupil dilation (PD) is an indirect and non‐exclusive marker of firing of the noradrenergic locus coeruleus (LC‐NA) system. The LC‐NA system undergoes both structural and functional decline in healthy and pathological aging. PD is known to increase with cognitive load or effort as well as an increased allocation of attentional resources. Findings on age‐related differences are mixed, with some evidence showing greater or more sustained pupil responses in older adults with increasing cognitive load, possibly reflecting compensatory mechanisms, while others show reduced or blunted responses. This study aimed to investigate whether changes in pupil size are associated with task‐dependent variations in performance in older and younger adults.

**Method:**

40 healthy younger adults (20‐30 years old) and 27 healthy older adults (60‐75 years old) completed three experimental tasks during eye‐tracking recordings. The tasks assessed different types of cognitive abilities and employed cube structure stimuli with varying levels of difficulty: a) a mental rotation tasks with varying levels of angle disparity; b) a perceptual decision task with varying levels of stimulus sizes; c) a working memory tasks with varying levels of memory load.

**Result:**

In both groups, behavioural performance was modulated by task difficulty with higher accuracy and faster responses in easier as compared to difficult conditions in all the tasks. Across all three tasks, older adults were slower and committed more errors than younger adults. Interestingly, age‐related differences in pupil dilation were detected only in the working memory task, where older participants showed greater pupil responses than younger participants. Furthermore, in the working memory tasks, pupil dilation was larger in difficult conditions.

**Conclusion:**

Our findings suggest that pupil dilation is not a general marker of cognitive effort but can be specifically sensitive to memory load. Moreover, the results are in line with the idea that larger PD may reflect age‐related compensatory mechanisms, occurring in a task‐specific fashion. In conclusion, the study offers novel hypotheses on the role of the LC‐NA system in cognitive function and its decline.